# eSNPO: An eQTL-based SNP Ontology and SNP functional enrichment analysis platform

**DOI:** 10.1038/srep30595

**Published:** 2016-07-29

**Authors:** Jin Li, Limei Wang, Tao Jiang, Jizhe Wang, Xue Li, Xiaoyan Liu, Chunyu Wang, Zhixia Teng, Ruijie Zhang, Hongchao Lv, Maozu Guo

**Affiliations:** 1 College of Bioinformatics Science and Technology, Harbin Medical University, Harbin, Heilongjiang, China; 2 School of Computer Science and Technology, Harbin Institute of Technology, Harbin, Heilongjiang, China; 3 School of Life Science and Technology, Harbin Institute of Technology, Harbin, Heilongjiang, China; 4 School of Basic Medical Sciences, Harbin Medical University, Harbin, Heilongjiang, China; 5 College of automation, Harbin Engineering University, Harbin, Heilongjiang, China

## Abstract

Genome-wide association studies (GWASs) have mined many common genetic variants associated with human complex traits like diseases. After that, the functional annotation and enrichment analysis of significant SNPs are important tasks. Classic methods are always based on physical positions of SNPs and genes. Expression quantitative trait loci (eQTLs) are genomic loci that contribute to variation in gene expression levels and have been proven efficient to connect SNPs and genes. In this work, we integrated the eQTL data and Gene Ontology (GO), constructed associations between SNPs and GO terms, then performed functional enrichment analysis. Finally, we constructed an eQTL-based SNP Ontology and SNP functional enrichment analysis platform. Taking Parkinson Disease (PD) as an example, the proposed platform and method are efficient. We believe eSNPO will be a useful resource for SNP functional annotation and enrichment analysis after we have got significant disease related SNPs.

Genome-wide association study (GWAS) is an examination of many common genetic variants in different individuals to see if any variant is associated with a trait. GWAS studies typically focus on associations between single nucleotide polymorphisms (SNPs) and traits like major complex diseases^1^. Since two SNPs with significantly altered allele frequency between the Age-related Macular Degeneration (ARMD) and healthy controls was firstly found in 2005^2^, more than 100,000 risk SNPs associated to hundreds of diseases in human have been mined via GWAS^3^. There are several GWAS databases for human diseases and traits, such as GWAS Catalog^3^, GWAS Central^4^ and GWASdb^5^,^6^.

After getting the significant SNPs, functional analysis is an important task. Generally, SNPs are considered to be functional through related genes, and the most popular method is SNP functional enrichment analysis. Gene ontology (GO) is a major bioinformatics initiative to unify the representation of gene and gene product attributes^7^,^8^. There are several SNP functional database, such as SNP Function Portal^9^ and F-SNP database^10^; and SNP functional enrichment analysis methods, such as I-GSEA4GWAS^11^, SNP-based pathway enrichment analysis^12^, SNPsnap^13^ and SNP2GO^14^. Similar to gene functional enrichment analysis, these methods can be divided into two categories, significant SNPs based methods and SNP sets based methods. A common ground in these methods is that the SNP functions are explained by the related genes according to physical positions on chromosome.

Expression quantitative trait loci (eQTLs) are genomic loci that contribute to variation in expression levels of mRNAs^15^. The first genome-wide gene expression QTL study was carried out in yeast and published in 2002^16^. Plenty of eQTL studies followed in plants and animals, including humans. Studies have shown that SNPs reproducibly associated with complex disorders are significantly enriched for eQTLs relative to frequency-matched SNPs^17^. Systematic integrations of eQTLs and GWAS have been used to identify risk genes in Schizophrenia^18^, Psoriasis^19^, and Muscle traits^20^. Therefore, eQTL data is an important and useful source for SNP functional annotation.

In this study, taking eQTL as medium between SNPs and their functions, we integrated eQTL and GO information and constructed a human SNP Ontology database and SNP functional enrichment analysis platform. It will be an efficient tool after GWAS analysis for a complex trait.

## Material and Methods

### eQTL data

The eQTL data were collected from several open databases and literatures. The gene expression patterns are specific among tissue types, and so do the eQTL patterns. Therefore, a classification by tissue types is necessary. We classified them into 12 tissues (Table 1). We combined the data from different studies of same tissue type. For each data, we set a significant threshold of FDR < 0.05. We retained only the SNPs with reference names and genes with gene symbols. In each tissue type, the numbers of samples, SNPs and genes are all after the screening.

### Brain data

As Parkinson Disease (PD) is a disorder of the central nervous system, we selected eQTL data in brain for a case study from Gibbs *et al*.^21^ and Myers *et al*.^22^. In Gibbs *et al*.’s study, four frozen tissue samples of the cerebellum (CRBLM), frontal cortex (FCTX), caudal pons (PONS) and temporal cortex (TCTX) were obtained from 150 neurologically normal Caucasian subjects resulting in 600 tissue samples. SNP genotyping was performed using Infinium HumanHap 550 beadchips (Illumina) for 561,466 SNPs. Profiling of 22,184 mRNA transcripts was performed using HumanRef-8 Expression BeadChips (Illumina). For each of the four brain regions, a regression analysis was performed using Plink^23^. After eQTL analysis in each brain regions, we integrated the results. In Myers *et al*.’s study, whole-genome genotyping for 366,140 SNPs and expression analysis of 14,078 genes were carried out on a series of 193 neurologically normal human brain samples using the Affymetrix GeneChip Human Mapping 500 K Array Set and Illumina HumanRefseq-8 Expression BeadChip platforms. A one-degree-of-freedom allelic test of association analysis was performed using Plink^23^. We integrated the results from these 2 studies. Finally, we got 51,131 significant correlations between 22,740 SNPs and 7,161 genes with the threshold of FDR < 0.05.

### Gene annotation data

The gene annotation data was downloaded from the Gene Ontology (GO) database (www.geneontology.org/page/download-annotations)^7^,^8^.

### ESNPO construction

**Figure 1:**
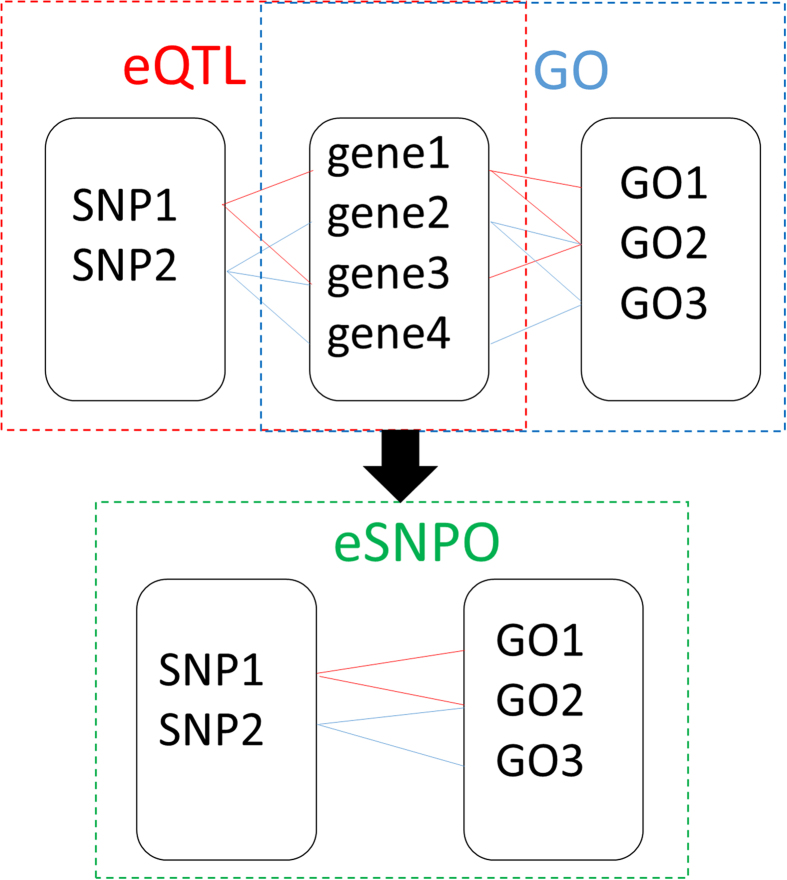
ESNPO construction.

We defined associations between SNPs and GO terms via combining the associations between SNPs and genes from eQTL and the associations between genes and GO terms from GO annotation database. A SNP and GO term with at least one common gene will be connected for an association. It was illustrated in Fig. 1.

### SNP functional enrichment analysis

We performed Fisher exact test to estimate the significance of associations between SNPs and GO terms. The Fisher exact test is equal to Hypergeometric test. Suppose there are N SNPs and M disease-related SNPs in eSNPO. For a given GO term, there are n SNPs and m disease-related SNPs. The *p* value is estimated as follows.


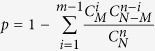


### P value adjustment

In an analysis, multiple GO terms are tested for significance and the Type I error would increase. Therefore, a multiple test adjustment is needed after estimating *p* values. There are 7 *p* value adjustment methods adopted using p.adjust function in R. The Bonferroni correction (“bonferroni”)^24^ in which the *p* values are multiplied by the number of comparisons. Less conservative corrections are also included by Holm (“holm”)^25^, Hochberg (“hochberg”)^26^, Hommel (“hommel”)^27^, Benjamini & Hochberg (“BH” or its alias “fdr”)^28^, and Benjamini & Yekutieli (“BY”)^29^, respectively. There is no golden standard to compare these methods, and the most popular method is False Discovery Rate method. The False Discovery Rate (FDR) is one way of conceptualizing the rate of type I errors in null hypothesis testing when conducting multiple comparisons. In this study, we used the “fdr” method.

## Database

After all, we construct a SNP Ontology and SNP functional enrichment analysis platform (http://bioinfo.hrbmu.edu.cn/esnpo/ or http://nclab.hit.edu.cn/esnpo/). It mainly includes 2 functions, eQTL-based SNP functional annotation and SNP functional enrichment analysis. After removing redundancy, we got 699,445 associations between 21,123 SNPs and 11,714 GO terms. The detailed statistics for the 12 tissues were illustrated in Table 2. The GO terms are formed by 3 components, Biological Process (BP), Cellular Component (CC) and Molecular Function(MF).

## Case study

### PD SNPs data

PD is a degenerative disorder of the central nervous system mainly affecting the motor system. We used 2,034 unique PD-related SNPs in Guiyou Liu *et al*.^30^. These SNPs came from these following works: 41 SNPs were from the GWAS Catalog^3^; 70 SNPs were from a large PD GWAS with over 3,400 cases and 29,000 controls conducted by Do *et al*.^31^; 783 SNPs were from a meta-analysis of PD GWAS with 4,238 PD cases and 4,239 controls performed by Pankratz *et al*.^32^; 1,292 SNPs were from a meta-analysis of PD GWAS using a common set of 7,893,274 variants across 13,708 cases and 95,282 controls conducted by Nalls *et al*.^33^. The threshold of *p* values in these studies were set to be 5.00E−08. After removing redundancy, we selected 2034 unique SNPs with *P* < 5.00E−08.

### PD enrichment analysis

In the eQTL-based SNP enrichment analysis, of the 2,034 SNPs, there are 846 SNPs annotated in 77 terms. After Fisher exact test, there are 67 (87.0%) significant terms under the threshold of *fdr* < 0.01.

In the position-based SNP enrichment analysis, of the 2,034 SNPs, there are 1,318 SNPs annotated in 807 terms. After Fisher exact test, there are 396 (49.1%) significant terms under the threshold of *fdr* < 0.01.

Compared between the significant results from eSNPO and position-based enrichment analysis, there are 43 terms in common, including 19 Biological Process (BP) terms, 14 Cellular Component(CC) terms and 10 Molecular Function (MF) terms.

From the results, though there are fewer annotated GO terms in eSNPO than position-based method, there are higher proportion of significant results in eQTL-based method.

To evaluate the method, we performed literature verification on these significant BP GO terms. Of these 19 BP terms in common between these 2 methods, there are 5 terms about axon or neurons; 5 terms about microtubule; 4 terms about apoptotic, cell death or autophagy; 1 term about pregnancy. The axon or neurons^34^,^35^, microtubule^36^,^37^,^38^, apoptotic^39^,^40^,^41^, cell death^42^,^43^ or autophagy^39^,^44^. pregnancy^45^,^46^ were verified by other studies.

Furthermore, we further verified these significant GO terms only obtained in eQTL-based method (8 BP terms, 8 CC terms and 8 MF terms). Of these 8 BP terms, there are 2 terms about apoptotic signaling pathway^47^, 1 term about cell proliferation^48^,^49^, 1 term about cell adhesion^50^, 2 term about JUN phosphorylation^51^ which have been verified by other studies.

## Conclusion

In this work, we constructed an eQTL-based SNP Ontology and SNP functional enrichment analysis platform (http://bioinfo.hrbmu.edu.cn/esnpo/ or http://nclab.hit.edu.cn/esnpo/). We integrated the eQTL data and GO, constructed associations between SNPs and GO terms, then performed functional enrichment analysis. Taking PD as an example, this eQTL-based method is an efficient method as the position-based method. Therefore, we believe it is a useful SNP functional enrichment analysis resource after we selected significant disease related SNPs.

However, there are still some shortages in this method. The first is there may not be enough suitable eQTL data we can use. And the second is that the scale of eSNPO is far less than the position-based method. These shortages will be solved along with more and more eQTL studies have been done.

## Additional Information

**How to cite this article**: Li, J. *et al*. eSNPO: An eQTL-based SNP Ontology and SNP functional enrichment analysis platform. *Sci. Rep.* **6**, 30595; doi: 10.1038/srep30595 (2016).

## Figures and Tables

**Table 1:** eQTL data in 12 tissues.

Tissue type	Samples	SNPs	Genes	Reference
Adipose Subcutaneous	111	18963	241	gtexportal^52^
Artery Tibia	124	28332	372	gtexportal^52^
Brain	765	22740	7161	eQTL Browser^53^, seeQTL^54^
Heart	87	14086	186	gtexportal^52^
Lung	124	31905	434	gtexportal^52^
Muscle Skeletal	143	25383	301	gtexportal^52^
Nerve Tibial	102	23253	327	gtexportal^52^
Skin	114	20506	296	gtexportal^52^
Blood	5479	406341	6780	Blood eQTL browser^55^, gtexportal^52^
Liver	427	2305	3463	eQTL Browser^53^
Lymphoblastoid	1220	208039	9168	eQTL Browser^53^, seeQTL^54^, Liming Liang^56^
Thyroid	112	33939	481	gtexportal^52^

**Table 2:** Summary statistics of eSNPO.

Tissue	SNPs	GO terms	BP	CC	MF
Adipose Subcutaneous	6168	304	166	75	63
Artery Tibial	8472	478	286	92	100
Brain	21123	11714	7979	1168	2567
Heart Left Ventricle	3049	294	171	59	64
Lung	8315	530	327	90	113
Muscle Skeletal	8637	501	318	96	87
Nerve Tibial	7210	533	334	89	110
Skin	4240	358	213	87	58
Blood	353817	11153	7514	1136	2503
Liver	1976	7762	5257	859	1646
Lymphoblastoid	184971	12158	8238	1174	2746
Thyroid	8258	637	385	113	139
